# Practices of decision making in priority setting and resource allocation: a scoping review and narrative synthesis of existing frameworks

**DOI:** 10.1186/s13561-020-00300-0

**Published:** 2021-01-07

**Authors:** Brayan V. Seixas, François Dionne, Craig Mitton

**Affiliations:** 1 Department of Health Policy and Management, Fielding School of Public Health, University of California, Los Angeles (UCLA), Los Angeles, USA; 2 Prioritize Consulting Inc., Vancouver, Canada; 3 Center for Clinical Epidemiology and Evaluation, Vancouver, Canada; 4 School of Population and Public Health, University of British Columbia (UBC), Vancouver, Canada

**Keywords:** Priority setting, Resource allocation, Frameworks, Health economics, Efficiency

## Abstract

**Background:**

Due to growing expenditures, health systems have been pushed to improve decision-making practices on resource allocation. This study aimed to identify which practices of priority setting and resource allocation (PSRA) have been used in healthcare systems of high-income countries.

**Methods:**

A scoping literature review (2007–2019) was conducted to map empirical PSRA activities. A two-stage screening process was utilized to identify existing approaches and cluster similar frameworks. That was complemented with a gray literature and horizontal scanning. A narrative synthesis was carried out to make sense of the existing literature and current state of PSRA practices in healthcare.

**Results:**

One thousand five hundred eighty five references were found in the peer-reviewed literature and 25 papers were selected for full-review. We identified three major types of decision-making framework in PSRA: 1) Program Budgeting and Marginal Analysis (PBMA); 2) Health Technology Assessment (HTA); and 3) Multiple-criteria value assessment. Our narrative synthesis indicates these formal frameworks of priority setting and resource allocation have been mostly implemented in episodic exercises with poor follow-up and evaluation. There seems to be growing interest for explicit robust rationales and ample stakeholder involvement, but that has not been the norm in the process of allocating resources within healthcare systems of high-income countries.

**Conclusions:**

No single dominate framework for PSRA appeared as the preferred approach across jurisdictions, but common elements exist both in terms of process and structure. Decision-makers worldwide can draw on our work in designing and implementing PSRA processes in their contexts.

## Introduction

Priority setting and resource allocation (PSRA) practices constitute processes and rationales applied to the pivotal task of determining how resources (i.e., money, people, time) are allocated within healthcare systems [1]. The volume of activity by type of intervention is not simply the result of the aggregation of individual clinical decisions but rather is predominantly the result of a budgeting decision. Given a pre-determined budget, decisions are made on the amount of funding budgeted for each type of intervention or area of care. Trade-offs then become a space not only for demonstration of clinical value but also of dispute for the scare resources. In essence, this is the practice of deciding what is covered and what is not, with the aim of reducing spending on low value activity.

A PSRA framework has at least two components: a mechanism to assess the value of interventions and a mechanism to guide the prioritization activity (i.e., making trade-offs). Other work has focused on approaches employed for the assessment of the value of health care interventions [2]. One common approach to value assessment is multi-criteria decision analysis (MCDA) [3, 4], which can be viewed as a competitor to the more traditional approach of the incremental cost effectiveness ratio (ICER) based on quality adjusted life years (QALYs) [5–8]. While it is critically important to understand value assessment, value assessment is not a choice making exercise per se. Value assessment needs hence to be set within a framework for decision making, i.e., the processual space and institutional environment where choices are made [9].

As previous research has indicated [10, 11], resource allocation in healthcare systems are often carried out based on historical decisions, without any explicit rationale and proper consideration of opportunity costs. Yet, the growing budgetary pressures observed in virtually every high-income country has led researchers and decision-makers to pursue novel methodologies to prioritize investment options and allocate the scarce existing resources. Thus, the present study aims to assess the evolution of the PSRA field understanding which practices have been developed and implemented.

Previous systematic reviews on PSRA are available in the literature, with distinct nuances. Some focus on priority-setting at the macro or meso levels [12], some focus on hospitals [13], and others emphasize frameworks implemented in low- and middle-income countries (LMIC) [12, 14]. The results are diverse and include a varied collection of case-studies. The distinct methodologies described in the literature as PSRA strategies have been employed in virtually every level of governance and type of healthcare setting. As each particular setting has specific contexts and goals, these decision-making approaches get different procedural formats and involve different stakeholders (for example, while a PSRA initiative may involve clinicians, administrators and other healthcare professionals within the realm of a single healthcare organization, a PSRA exercise may involve legislators, bureaucrats and representatives of the general public at a government level). In the realm of publicly funded healthcare systems, the PSRA initiatives play a vital role helping decision-makers to improve budgetary and financial management, ensuring legitimacy, fairness and transparency while also adding value to decisions on resource allocation [15, 16]. Effective public financial management depends on explicit and formal PSRA approaches [17, 18].

The current study focuses on existing mechanisms, processes or frameworks to guide prioritization. Specifically, the objective was to identify frameworks that have been employed in real-world settings in high-income countries. Our intent was not to identify every single implementation of a given framework but rather to report on instances where key frameworks were utilized. We do not intend to provide a meta-analysis of all available evidence, but rather touch upon the most relevant aspects for reporting a collection of frameworks used in practice for priority setting and resource allocation decision-making across countries. A variety of methodologies have been described in the health economics literature under the PSRA umbrella terminology. They may have markedly different procedures and rationales, such as Health Technology Assessment (HTA) and Programme Budgeting and Marginal Analysis (PBMA). However, as long as they are used to assess the value of existing investment alternatives and to guide the choice-making process, they have been deemed PSRA strategies in the literature. Given that our goal is to map the literature to identify existing frameworks with some evidence of empirical use, we did not challenge authors in their categorization of any given methodology as a PSRA framework.

We narrowed down the analysis for high-income countries mainly because the urge for efficient resource allocation in these settings is a response to a critical historical trend of unsustainable growth of health expenditures, which makes the motivation and goal very different from LMICs, whose health systems are often underfunded and where PSRA strategies have the objective of achieving universal healthcare coverage. In addition, a recent review was conducted in LMIC countries [14] and turned up very limited empirical applications which was our focus here.

The overall research question guiding this study was: which decision-making frameworks have been developed and implemented to set priorities and allocate resources within healthcare systems of high-income countries? To answer this question, we conducted a scoping review of the peer-reviewed scientific literature, a gray literature review and horizontal scanning, and then a narrative synthesis to make sense of obtained data and dialogue with other pieces from the literature.

## Methods

A scoping review was conducted focusing on frameworks used for PSRA in high-income countries. A comprehensive search of the peer-reviewed literature published between 2007 and 2019 was conducted using Ovid MEDLINE, an extensive database of public health journals with a platform for building searching strategies. The literature review search strategy is outlined in Appendix 1. Given that the study objective was to identify frameworks with actual value for implementation in healthcare systems (and not simply a historical view of the field), searching older papers would be limited in describing current practice and also would overlap with previous studies.

**Fig. 1:**
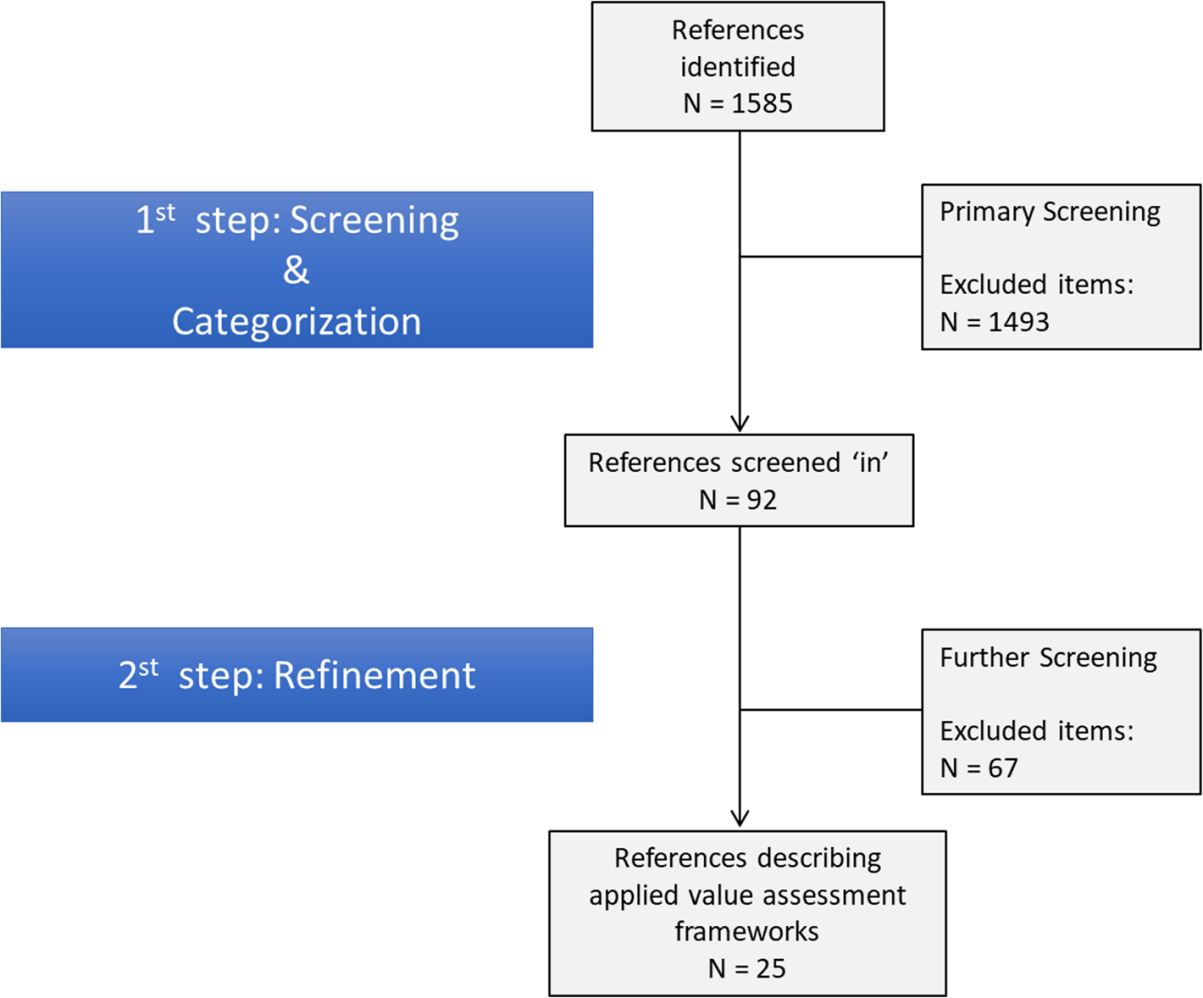
Screening 115 steps resulting in final full-text article review.

In total, 1585 titles and abstracts were found, once duplicates were removed. We then used a two-step screening process. First, the 1585 abstracts were screened by one primary reviewer and two secondary reviewers based on the inclusion/exclusion criteria found in Appendix 2. Reviewer 1 (BVS) reviewed all 1585 articles while reviewer 2 (CM) reviewed 227 articles and reviewer 3 (FD) reviewed 71 articles. The agreement rate between reviewer 1 and reviewers 2 and 3 was over 90%. Discrepancies were handled conservatively, resulting in a total of 92 abstracts initially being screened ‘in’. Following this, one of the two senior reviewers (CM) took a further detailed read of the abstracts and pared the list down to 25 relevant articles. This second stage screen in the main excluded papers that initially appeared to be an empirical study but were in fact discussing some aspect of priority setting without an actual case study or implementation of a framework. We then applied a data extraction tool (see Appendix 3) to identify the relevant information. Figure 1 presents a step flow diagram depicting the screening process.

Note that because of the objective and design of this review (which focuses on revealing existing formal PSRA frameworks, thus including only papers that provided a full description of a framework employed in a real-world setting), it is likely that articles presenting relevant information on current practices of decision-making in priority-setting have not been captured.

Thus, we also conducted horizontal scanning and conducted searches in the gray literature. The major search strategy here consisted of exploring the websites of reputable HTA agencies and other relevant international organizations for presentations, guidelines, working papers or any other pertinent piece of gray literature. We looked at the following organizations: Health Technology Assessment International (HTAi); International Network of Agencies for Health Technology Assessment (INAHTA); International Society for Pharmacoeconomics and Outcomes Research (ISPOR); European Network for Health Technology Assessment (EUnetHTA); International Health Economics Association (IHEA); Agency for Health Research and Quality (AHRQ); Canadian Agency for Drugs and Technologies in Health (CADTH); Kaiser International Health Group; and Blue Cross Blue Shield Association. Furthermore, we also perused other existing literature reviews and their references in order to obtain a deeper and richer view of PSRA field. These documents were not included in our primary data analysis as they were not empirical studies per se but provide important context and further insight with respect to PSRA.

As discussed in the Introduction, a PSRA framework constitutes a formal process to determine the available options for investment and a rationale for choice making. These frameworks can be applied at any level of governance or type of healthcare organization. No further a priori criteria were defined to determine whether or not a study constitutes a PSRA initiative, allowing categories to emerge from the data. In other words, we did not aim to challenge previously published authors in their judgment on whether or not certain approaches constitute PSRA frameworks, but rather we aimed to document what has been done in the field.

In addition, we focused on describing the set of existing PSRA frameworks rather than reporting all the instances a given methodology is employed. We ultimately aim to provide a helpful overview of available practices for decision-makers and those interested in the process of priority setting and resource allocation in health care.

## Results

The 25 papers that met the inclusion criteria and were subject to data extraction provide relevant information from ten countries: Australia [19–21]; Austria [22]; Canada [23–27]; Israel [28]; Korea [29, 30]; New Zealand [31]; Norway [32]; Sweden [33, 34]; UK [35–42]; US [43]. The list of frameworks identified in our view provides a reasonable summary of PSRA frameworks developed and implemented in health care systems in high-income countries.

### Emerging classification system

Based on the information obtained through our extraction tool (Appendix 3), three major umbrella categories emerged to make sense of possible grouping similarities among the identified PSRA initiatives: 1) PBMA frameworks; 2) HTA-related frameworks; and 3) multi-criteria value assessment frameworks. Before we delve into how the identified practices fit these categories, let us better define each classification category.

First, Program Budgeting and Marginal Analysis (PBMA) is a deliberative framework used to assist decision-makers in determining what to fund and what not to fund. It can be used to achieve specific goals (such as deal with existing budgetary deficits or direct resources to capital investments) or as a routine practice of decision making in resource allocation. PBMA provides the process and structure within which a specific value assessment approach can be applied. The steps of the process are usually as follows [44]: 1) determine the aim and scope of the priority setting exercise; 2) examine how resources are currently spent; 3) form a multi-disciplinary committee to identify the relevant decision criteria; 4) identify proposals for changes to the current spending pattern, either for investment (increased spending) or disinvestment (reduced spending), and in all cases the focus is on marginal analysis or change to the status quo; 5) the impact of each proposals is then assessed by the committee using the pre-identified criteria; 6) make decisions based on relative value trade-offs; 7) provide an opportunity for appeal based on pre-defined guidelines; and 8) evaluate the process and make adjustments to refine the process as necessary. The process steps are made known throughout the organization and key stakeholders are involved in proposal generation as much as possible. Public members or patient representatives can also be engaged to provide input on the relevant criteria or the criteria weights. The chosen criteria and their respective weights typically vary across jurisdictions, reflecting local values and preferences. A PBMA framework can be applied at virtually any organizational setting, e.g., a system level, an individual hospital or a unit level within a given hospital.

Second, Health Technology Assessment (HTA) is formally defined by INAHTA as “the systematic evaluation of the properties and effects of a health technology, addressing the direct and intended effects of this technology, as well as its indirect and unintended consequences, and aimed mainly at informing decision making regarding health technologies” [45]. Similarly, the European Network for Health Technology Assessment states that “HTA is a multidisciplinary process that summarises information about the medical, social, economic and ethical issues related to the use of a health technology in a systematic, transparent, unbiased, and robust manner” [46] Note that this type of endeavor is distinguished from priority-setting, which is the choice making activities that decision makers undertake in determining what health care services to fund and what not to fund [47]. The purpose of HTA per se is not to realize or implement trade-offs, pointing out investment and disinvestment opportunities [48]. Rather, HTA in itself is a tool to produce evidence that helps inform the management of technologies. Harris et al. [49] state that HTA is a valuable tool for decision making and its use may lead to disinvestment but it is not a framework specifically intended for assessing trade-offs, broadly speaking. It is possible to conceive, however, a broader framework of priority setting predominantly based on HTA.

Third, the last category of priority-setting framework emerged directly from the data analysis of identified empirical PSRA activities and refers to organization-wide priority-setting processes with explicit use of multiple criteria for value assessment. Under this category some sort of explicit and formal consideration of multiple criteria was carried out, which could be a formal MCDA (multi-criteria decision analysis) tool, a discrete-choice methodology, or some other deliberative process involving criteria assessment. Thus, this category encompasses a wide variety of practices that ultimately have one thing in common: the consideration of multiple criteria and participation of multiple stakeholders in the priority-setting decision-making process. Of note, PBMA frameworks often use MCDA tools and thus could fall under this catch-all category of frameworks that lead to choices based on multiple criteria. Yet, because of its predominance in the literature and its particular underpinning principles, we have chosen herein to refer to PBMA as its own category. Also, the definition of value seems to vary among these studies and was rarely provided.

### Classification of identified studies

As it can be seen in Table 1, out of the 25 studies, nine were found to have used a formal PBMA framework, three employed an HTA-related framework, and thirteen were broadly classified as multiple-criteria value assessment framework. We do not at all claim that these are the full set of applications of PSRA. Rather, this list reflects current practice and is likely representative of the types of approaches in use at this time.

**Table 1:** Practices of priority setting found in the literature

Authors	Date	Country	Level of exercise	Area of application	Primary decision maker	Classification category
Peacock et al. [19]	2007	Australia	Regional	Mental health services	Executive team	PBMA framework
Galego et al. [20]	2007	Australia	Hospital	Drugs	Executive team	Multi-criteria value assessment framework
McDonald et al. [21]	2011	Australia	Regional	Primary care services	Executive team	Multi-criteria value assessment framework
Mentzaskis et al. [22]	2014	Austria	National	General health services	Policy makers	Multi-criteria value assessment framework (specifically, DCE)
Urquhart et al. [23]	2008	Canada	Regional	Home and community care	Executive team	PBMA framework + A4R
Dionne et al. [24]	2009	Canada	Regional	General health services	Executive team	PBMA framework
Stafinski et al. [25]	2011	Canada	National	Health technologies	Policy makers	HTA-related framework
Mitton et al. [26]	2011	Canada	Regional	Primary care, community care and public health	Executive team	PBMA framework
Cornelissen et al. [27]	2016	Canada	Regional	Community care	Executive team	PBMA framework
Greenberg et al. [28]	2009	Israel	National	Health technologies	Policy makers	HTA-related framework
Ahn et al. [29]	2012	Korea	National	Health technologies	Policy makers	HTA-related framework
Choe et al. [30]	2014	Korea	National	Vaccines	Policy makers	Multi-criteria value assessment framework
Ashton et al. [31]	2008	New Zealand	Regional	General health services	Executive team	Multi-criteria value assessment framework
Defechereux et al. [32]	2012	Norway	National	General health services	Policy makers	Multi-criteria value assessment framework
Waldau et al. [33]	2010	Sweden	Regional	General health services	Executive team	Multi-criteria value assessment framework + A4R
Waldau et al. [34]	2015	Sweden	Regional	General health services	Executive team	Multi-criteria value assessment framework + A4R
Bate et al. [35]	2007	UK	Regional	Orthopedic surgery	Executive team	PBMA framework
Wilson et al. [36]	2007	UK	Regional	General health services	Executive team	PBMA framework
Airoldi et al. [37]	2008	UK	National	Diabetes	Policy makers	Multi-criteria value assessment framework
Marsh et al. [38]	2013	UK	National	Preventative health interventions	Policy makers	Multi-criteria value assessment framework
Goodwin et al. [39]	2013	UK	Regional	General health services	Executive team	PBMA
Airoldi et al. [40]	2013	UK	Regional	Mental health services	Executive team	Multi-criteria value assessment framework
Holmes et al. [41]	2018	UK	Regional	Dental services	Executive team	PBMA
Vernazza et al. [42]	2019	UK	Regional	Dental services	Executive team	Multi-criteria value assessment framework
Canham-Chervak et al. [43]	2010	US	National	Military injuries	Executive team	Multi-criteria value assessment framework

One study falling within the PBMA framework category and two falling within multiple-criteria value assessment framework explicitly stated applying Accountability for Reasonableness (A4R) as well. Sometimes referred to in the literature as a priority-setting framework, A4R consists of a set of principles developed by Daniels and Sabin [50, 51] focusing on the ethical aspects of choice-making. The underlying thinking is that there is inescapable uncertainty in decision making in health care and therefore it is critical to the acceptability of the decisions that the prioritization process be perceived as a fair process by the stakeholders. The original framework had four elements or conditions that determine the perceived fairness of a given process: relevance, publicity, revision and enforcement. Gibson and colleagues [52] then proposed a fifth condition: empowerment. A4R can essentially be used with any approach to decision making in that, however the decisions are made on resource allocation, the ethical conditions of A4R can be brought to bear to ensure that those decisions are made as fairly as possible.

### Key findings and further contextual comments

We found that formal PSRA frameworks have been used in virtually all possible levels of governance and administration (national, state/provincial, regional, hospital) and have served for the prioritization of a wide variety of health services (e.g., from community care, to mental health, drug reimbursement, immunization and specific diseases). Despite this heterogeneity in application, a number of key process characteristics were identified from the selected papers:

- PSRA strategies have employed a variety of criteria to assess value, and do not necessarily use a single, consistent technique to judge alternatives and summarize preferences;
- A variety of stakeholders were involved in almost every case, such as administrators, government officials and clinicians;
- Decisions were typically not subject to review by external stakeholders (i.e., the general public);
- It was generally not clear from the papers how requests for funding were initiated, nor was it specified if current spending was reviewed as part of the process of decision making (although PBMA, for example, implies such a review);
- Several types of data were reported to inform decision-making, including published literature, clinical opinions, economic evaluations, HTAs, and data on disease prevalence;
- There was limited reporting of evaluation in these studies;
- Types of information reported to be drawn on for decision making included published literature, clinical opinions, economic evaluations and data on disease prevalence;
- Some level of political involvement was stated in most cases, although in cases where a more robust framework is described, there seemed to be less political interference;
- Deliberation has largely become the norm, e.g.: “following common practice in decision analysis, validity and consistency of responses was established through panel discussion and deliberation” [19], p. 908];
- It seems that approaches of willingness-to-pay thresholds are being abandoned in light of a greater understanding of the complexities of health care decision making, of the limitations of ‘single truth’ evidence and of the need for broader stakeholder engagement.

Note that because of the objective and design of this review, which focused on revealing existing formal PSRA frameworks that were employed in a real-world setting, it is a certainty that relevant articles presenting information on current practices of decision-making in priority-setting were not captured (e.g., think pieces or other non-empirical activity). To reiterate, the purpose was not to capture every framework that has been employed, and in every situation, but rather to provide an indication of key frameworks that have been applied with some consistency across countries.

An important contextual factor is that the majority of PSRA initiatives were found in countries where there is a pre-set limit on how much can be spent and the organizations holding the envelope must find ways to stay within this limit. That is, the total value of services provided over a year is largely determined at the outset as a fixed envelope and providers have to prioritize anticipated claims or adjust the fee structure (reimbursement level) in order to keep total costs within the envelope. Defechereux et al. [32] summarize the nature of this challenge: “In all health care systems, choices in the allocation of resources are necessary. Public resources ( …) are insufficient to provide all possible services”.

Whereas priority-setting approaches based on decision sciences have been shown to be useful and versatile in allocating scarce resources in a wide variety of levels of governance and administration (hospital, regional and national levels [53]) as well as within diverse areas of care (like mental health [54], coronary heart diseases [55], and community care [27]), approaches grounded on welfare economics have been usually employed only to make recommendations regarding the coverage of specific technologies. Cost-effectiveness analysis and cost-utility analysis have been widely used within this restricted context of ‘priority-setting’ by HTA agencies all across the globe, such as CADTH in Canada, PBAC and MSAC in Australia, PHARMAC in New Zealand, and NICE in the UK.

Additional PSRA frameworks with some evidence of empirical use are also observed in the literature. Angelis and Kanavos [56], for instance, propose a MCDA-based approach called Advanced Value Framework, which uses five domains of criteria (burden of disease, therapeutic impact, safety profile, innovation level and socioeconomic impact) and a MAVT function to aggregate scores. Another framework based on MCDA that was developed to inform decision-making in health care and has been proposed for priority-setting and resource allocation is EVIDEM [57]. Airoldi et al. [58] propose the Socio-Technical Allocation of Resources (STAR) for resource allocation, as it is claimed to be theoretically strong and highly useful for decision-makers. STAR employs models to appraise the “cost-effectiveness of all interventions considered for resource reallocation by explicitly applying the theory of health economics to evidence of scale, costs, and benefits, with deliberation facilitated through an interactive social process of engaging key stakeholders” [58]. In this so-called ‘social process’, the involved stakeholders produce missing estimates of scale, costs, and benefits of the interventions, create visual representations of their relative cost-effectiveness and then interpret them. STAR was used by a Primary Care Trust (a local NHS planning agency) to allocate a fixed budget in 2008 and 2009 [58].

## Discussion

Our work found 25 studies describing a real-world practice of a formal framework of priority setting and resource allocation in ten high-income countries. In the process of making sense of all qualitative data generated by the scoping review through our previously designed extraction tool, we created a classification system that grouped identified studies in three categories: PBMA framework, HTA-related framework, and multi-criteria value assessment framework. Unlike the first two categories that refer to studies with explicit mention to PBMA and HTA components, the last category had some residual component and had a loose common feature, i.e., the use of multiple criteria and multiple stakeholder involvement in the process of decision-making (which could involve a formal MCDA tool or a DCE, for example). Although PBMA and HTA have clearly different goals and rationales, both approaches have been paradigmatically deemed frameworks of priority setting and resource in the health economics and health policy literature [1, 11–13, 59].

This study contributes to the literature not only in identifying which formal strategies of priority setting and resource allocation have been developed and implemented in healthcare systems of high-income countries, but also reveals important issues for the field of health economics and health policy. First, it indicates that formal decision-making processes with explicit and legitimate rationales are seemingly still episodic and have not turned into routine practice. Second, it reveals that although several important initiatives have been tried, evidence from evaluation is rare and there is still much to be learned about which practices are more successful and which system characteristics might be associated with them. Third, our findings suggest that the conventional extra-welfarist position that supports the mechanistic employment of a single value measure like the incremental cost-effectiveness ratio (ICER) has been losing space in favor of decision-making approaches that incorporate multiple criteria and combine multiple actors’ views. Fourth, a broad set of types of evidence is being used, moving beyond the traditionally gold-standard randomized controlled-trial and even peer-reviewed observational studies towards incorporating experts’ opinions and patients’ perspectives. Fifth, we noticed that appeals mechanism or review process for final decisions, a key element within A4R, are virtually absent from the empirical strategies of priority setting and resource allocation.

In a review focusing on resource allocation and disinvestment, Polisena et al. [11] found 14 studies, all in high-income countries. Two of them reported use of HTA to propose disinvestments whereas the majority described applications of PBMA. Studies reported initiatives at the national level (basically the HTA approaches towards disinvestment), at the regional level (health authorities) and at a single health care unit or department. Another review carried out by Barasa et al. [13] with particular interest in formal PSRA initiatives in hospital settings revealed a small number of studies, which were mostly based in high-income countries. Almost all of these exercises addressed allocation of resources among hospital departments (usually based on PBMA or MCDA) or decision-making regarding acquisition of specific technologies (employing CEA/CUA).

Hipgrave et al. [12] and Wiseman et al. [14] conducted systematic reviews emphasizing PSRA endeavours in low- and middle-income countries. They both point out that relatively little information is known about practices of decision-making in priority-setting within health care systems of developing countries in comparison to high-income settings. The majority of reports identified by Wiseman et al. [14] involve global or regional efforts of Global Cost-effectiveness Analysis (GCEA) using cost per DALY averted, as the WHO frameworks to identify the most cost-effective interventions to achieve the Millennium Development Goals and Universal Coverage. In almost all of these cases, options for disinvestment were not considered alongside options for investment. Both reviews identified ranking of alternatives based on MCDA tools, including initiatives in Ghana and Nepal. In addition, other approaches were identified, such as case-studies of multi-criteria frameworks based on A4R in Tanzania and Uganda and the applications of the Investment Case approach in India, Indonesia and Philippines.

In another review of the published literature conducted by Cromwell et al. [10] to find examples of ‘real-world’ priority setting exercises that used explicit criteria to guide decision-making, several case-studies were identified, mainly in Canada and UK. The most common approaches identified were PBMA and MCDA applied in various settings, e.g., national level, health authorities, hospitals and for specific disease programmes. A range of criteria were identified, with effectiveness and equity appearing most often.

Relying upon the evidence found in previous literature reviews on priority-setting, Hipgrave et al. [12] comments that “the overarching conclusion was that even in high-income settings where participatory, accountable and rational approaches to health priority-setting should be achievable, the process and outcomes of such exercises have been unsatisfactory”. The evidence from the published literature is usually about specific case-studies and very rarely report a systematic and continuous use of formal PSRA frameworks. A 2017 review [59] aiming to understand ‘how have systematic priority setting approaches influenced policy making’ concludes that “while systematic approaches have the potential to improve healthcare priority setting; most have not been adopted in routine policy making”. Having said that, Tsourapas and Frew [60] found that PBMA applications specifically have shown much success across countries and particularly in Canada. Thus, it seems that, as it is often the case, individual details on implementation and indeed the individuals involved play a key part in achieving success or not.

Our review has a few limitations. First, as it focuses on high-income countries, we might have failed to capture some interesting and auspicious approaches being performed elsewhere. Yet, given the limited existing literature on PSRA frameworks in other settings, and our own knowledge of the field over the last 20 years, that is unlikely to be the case. Second, due to the hues of grounded theory in analyzing the massive amount of qualitative data found in the literature review (as no rigid and formal a priori analytical framework was used to categorize and critique the practices deemed as priority setting and resource allocation initiatives), the boundaries among the frameworks are not always so clear. Similarly, their definition as a type of PSRA endeavor can be debatable, as opposed to a value assessment framework, for example. These and other possible inconsistencies are not due to our analysis, rather it is a manifestation of a broad literature that not always operates with clear and robust theoretical underpinning ideas around priority setting and value assessment. Third, the review is limited to existing empirical initiatives, which means that it is possible that promising and insightful theoretical frameworks have been left out. Lastly, our review has been limited to works published in the English language. It is possible that some relevant work had been captured by our search strategy for that reason.

## Conclusion

The unsustainable growth of health expenditures in high-income countries has led researchers and decision-makers to pursue efficiency in managing existing resources. The present work sought to identify which formal decision-making frameworks of priority setting and resource allocation have been developed and implemented in healthcare systems. We found three major categories of initiatives in this realm: PBMA, HTA and other multiple-criteria value assessment frameworks. Most were presented as an episodic management exercise, lacking information on evaluation and further implementation in routine practice.

In terms of future research, our work indicates a few important areas for further exploration. First, the epistemological boundaries between priority setting, value assessment and health technology assessment are not always clear. There seems to be space for a robust and extensive theoretical work aiming to establish these definitions in an interactive way, determining the nature of each endeavor with an explicit reference to the ontological frontiers that delimitate them. Such epistemological enterprise would have to be conducted with a clear view of its operational implications, in terms of practices and institutions. Second, more emphasis should be put on evaluation of implemented practices of decision-making. Very few studies present and discuss evaluation findings. This points out not only to the need of more focus on evaluating the existing PSRA practices but also to the need of developing novel evaluative tools in this realm. Third, as most papers present case-studies of PSRA initiatives that were implemented for a particular purpose (and usually not even evaluated), it is of high importance to establish a converging agenda for the development of PSRA frameworks that can be turned into routine processes. As virtually every health care organization is making decisions on what to fund and what not to fund, PSRA is most certainly happening but this is very often done in non-explicit and informal manners. Ensuring decisions are consistently made on reasonable, formal and agreed bases is expected to result in more efficient, equitable and legitimate allocation of the scarce resources available.

## Data Availability

Data are publicly available.

## Appendix 1

**Table 2:** Literature Review Search Strategy

**Database:** MEDLINE (OVID)	
Ovid MEDLINE(R) In-Process & Other Non-Indexed Citations & and Ovid MEDLINE(R) < 1946 to Present>	
**Search Name:** 2017 Res M10	
**Date:** Nov.18, 2017	
**Search Strategy:**	
1 (framework or frameworks).tw,kw. (213264)	
2 (tool or tools).tw,kw. (588532)	
3 case stud$.mp. (89455)	
4 (approach or approaches).tw,kw. (1447206)	
5 or/1–4 [Frameworks] (2153708)	
6 *resource allocation/ (3486)	
7 *health care rationing/ (6485)	
8 *health priorities/ (5178)	
9 or/6–8 (13437)	
10 5 and 9 (2152)	
11 comment/ or editorial/ or letter/ or news/ (1858162)	
12 10 not 11 (2083)	
13 limit 12 to yr = “2007 -Current” (1077)	
14 limit 13 to English language (998)	
15 commissioning.mp. (3703)	
16 5 and 15 (923)	
17 comment/ or editorial/ or letter/ or news/ (1858162)	
18 16 not 17 (911)	
19 limit 18 to yr = “2007 -Current” (723)	
20 limit 19 to English language (718)	
21 20 not 14 (707)	

## Appendix 2

**Table 3:** Inclusion and Exclusion Criteria of Scoping Literature Review

Inclusion Criteria	Exclusion Criteria
Empirical study or exercise of priority setting in so far as it involves choice making at any level in the health system (e.g. national, provincial/state, regional, single organization)	Priority setting for non-health care settings (animal, environmental, education, etc.)
	Exercises that involve priority setting for health research
	Reviews, commentaries or think pieces (although they may be kept for broader context)
	Studies in low- or middle-income country (LMICs)
Presentation of an actual framework for decision making in relation to priority setting (e.g., CEA in and of itself, or MCDA in and of itself, does not constitute a framework for priority setting)	Procurement, supply management, other purely financial mechanisms for cost containment
	Descriptions of bedside or strictly clinically focused priority setting/rationing, including organ donation
	Descriptions of only a single aspect of priority setting, even if empirically focused (e.g. public engagement, evaluation activity) where the whole process or framework is not described

## Appendix 3

**Table 4:** Extraction Tool

IDENTIFICATION	
1. In what country did the implementation take place?	
2. What level or part of the system did the priority setting implementation take place? (e.g., national, provincial, regional, hospital, community care, etc.)	
3. What was the scope or context of the implementation? (e.g., drugs, vaccines, disease area, across disease areas, across sectors, etc.)	
DECISION-MAKING	
4. Who establishes the strategic guidance for the organization and how specific is it (i.e. how much room is there for interpretation)?	
5. How does the organization establish priorities and make decisions on where to increase or reduce spending?	
• Is there any formal process or framework that is used for that purpose or is it done on a case by case basis?	
• How are requests for funding initiated? Who do they go to?	
• Is current spending typically reviewed as part of the process?	
• Are the organization’s priority setting decisions subject to review by external stakeholder(s)?	
6. What stakeholders were involved in the decision-making process? (e.g., researchers, policy makers, public members, patients, clinicians)	
7. What types of evidence/information are taken into account? (e.g., epidemiological evidence, clinical evidence, economic evidence, expert opinion, patient reported outcomes)	
EVALUATION	
8. Was there a reported discrepancy between ‘recommendations’ and actions taken?	
9. Was the implementation formally evaluated and if so what were the findings of the evaluation (specifically, were health outcomes impact assessed)? How successful was the implementation? What are the key lessons learned?	
10. What was the level of political involvement? Were notions of equity explicitly considered?	

## Abbreviations

A4R: Accountability for Reasonableness
AHRQ: Agency for Health Research and Quality
CADTH: Canadian Agency for Drugs and Technologies in Health
CEA: Cost-Effectiveness Analysis
CUA: Cost-Utility Analysis
EUnetHTA: European Network for Health Technology Assessment
HTA: Health Technology Assessment
HTAi: Health Technology Assessment International
IHEA: International Health Economics Association
INAHTA: International Network of Agencies for Health Technology Assessment
ISPOR: International Society for Pharmacoeconomics and Outcomes Research
MCDA: Multi-Criteria Decision Analysis
NHS: National Health Service (United Kingdom)
NICE: National Institute for Health and Care Excellence
PBAC: Pharmaceutical Benefits Advisory Committee
PBMA: Program Budgeting and Marginal Analysis
PSRA: Priority Setting and Resource Allocation
QALY: Quality-Adjusted Life Years
RCT: Randomized Controlled Trial

## References

[1] Mitton C, Donaldson C (2009). Priority Setting Toolkit : Guide to the Use of Economics in Healthcare Decision Making (1).

[2] Seixas BV, Dionne F, Conte T, Mitton C (2019). Assessing value in health care: using an interpretive classification system to understand existing practices based on a systematic review. BMC Health Serv Res.

[3] Marsh K, Lanitis T, Neasham D, Orfanos P, Caro J (2014). Assessing the value of healthcare interventions using multi-criteria decision analysis: a review of the literature. PharmacoEconomics.

[4] Thokala P, Devlin N, Marsh K, Baltussen R, Boysen M, Kalo Z (2016). Multiple criteria decision analysis for health care decision making—an introduction: report 1 of the ISPOR MCDA emerging good practices task force. Value Health.

[5] Diamantopoulos A, Sawyer LM, Lip GYH, Witte KK, Reynolds MR, Fauchier L (2016). Cost-effectiveness of an insertable cardiac monitor to detect atrial fibrillation in patients with cryptogenic stroke. Int J Stroke.

[6] Stout NK, Lee SJ, Schechter CB, Kerlikowske K, Alagoz O, Berry D (2014). Benefits, harms, and costs for breast cancer screening after US implementation of digital mammography. J Natl Cancer Inst.

[7] Bristow RE, Santillan A, Salani R, Diaz-Montes TP, Giuntoli RL, Meisner BC (2007). Intraperitoneal cisplatin and paclitaxel versus intravenous carboplatin and paclitaxel chemotherapy for stage III ovarian cancer: a cost-effectiveness analysis. Gynecol Oncol.

[8] Harron K, Mok Q, Dwan K, Ridyard CH, Moitt T, Millar M (2016). CATheter Infections in CHildren (CATCH): a randomised controlled trial and economic evaluation comparing impregnated and standard central venous catheters in children. Health Technol Assess.

[9] Mitton C, Donaldson C (2004). Health care priority setting: principles, practice and challenges. Cost Eff Resour Alloc.

[10] Cromwell I, Peacock SJ, Mitton C (2015). ‘Real-world’ health care priority setting using explicit decision criteria: a systematic review of the literature. BMC Health Serv Res.

[11] Polisena J, Clifford T, Elshaug AG, Mitton C, Russell E, Skidmore B (2013). Case studies that illustrate disinvestment and resource allocation decision-making processes in health care: a systematic review. International journal of technology assessment in health care. Cambridge..

[12] Hipgrave DB, Alderman KB, Anderson I, Soto EJ (2014). Health sector priority setting at meso-level in lower and middle income countries: lessons learned, available options and suggested steps. Soc Sci Med.

[13] Barasa EW, Molyneux S, English M, Cleary S (2015). Setting healthcare priorities in hospitals: a review of empirical studies. Health Policy Plan.

[14] Wiseman V, Mitton C, Doyle-Waters MM, Drake T, Conteh L, Newall AT (2016). Using economic evidence to set healthcare priorities in low-income and lower-middle-income countries: a systematic review of methodological frameworks. Health Econ.

[15] Allen R, Schiavo-Campo S, Garrity TC. Assessing and Reforming Public Financial Management: The World Bank; 2003. [cited 2020 Dec 14]. Available from: https://elibrary.worldbank.org/doi/abs/10.1596/0-8213-5599-6.

[16] Kioko SN, Marlowe J, Matkin DST, Moody M, Smith DL, Zhao ZJ (2011). Why public financial management matters. J Public Adm Res Theory.

[17] Public expenditure management handbook. [Internet]. [cited 2020 Dec 14]. Available from: https://elibrary.worldbank.org/doi/pdf/10.1596/0-8213-4297-5.

[18] Alkaraan F (2018). Public financial management reform: an ongoing journey towards good governance. J Financ Rep Account.

[19] Peacock SJ, Richardson JRJ, Carter R, Edwards D (2007). Priority setting in health care using multi-attribute utility theory and programme budgeting and marginal analysis (PBMA). Soc Sci Med.

[20] Gallego G, Taylor SJ, Brien JE (2007). Priority setting for high cost medications (HCMs) in public hospitals in Australia: a case study. Health Policy.

[21] McDonald J, Ollerenshaw A (2011). Priority setting in primary health care: a framework for local catchments. Rural Remote Health.

[22] Mentzakis E, Paolucci F, Rubicko G (2014). Priority setting in the Austrian healthcare system: results from a discrete choice experiment and implications for mental health. J Ment Health Policy Econ.

[23] Urquhart B, Mitton C, Peacock S (2008). Introducing priority setting and resource allocation in home and community care programs. J Health Serv Res Policy.

[24] Dionne F, Mitton C, Smith N, Donaldson C (2009). Evaluation of the impact of program budgeting and marginal analysis in Vancouver Island health authority. J Health Serv Res Policy.

[25] Stafinski T, Menon D, McCabe C, Philippon DJ (2011). To fund or not to fund. Pharmacoeconomics..

[26] Mitton C, Dionne F, Damji R, Campbell D, Bryan S. Difficult decisions in times of constraint: Criteria based Resource Allocation in the Vancouver Coastal Health Authority: University of British Columbia; 2011. [cited 2017 Nov 5]. Available from: https://open.library.ubc.ca/cIRcle/collections/facultyresearchandpublications/52383/items/1.0223748. 10.1186/1472-6963-11-169 PMC3155484 21756357

[27] Cornelissen E, Mitton C, Davidson A, Reid C, Hole R, Visockas A-M (2016). Fit for purpose? Introducing a rational priority setting approach into a community care setting. J Health Organ Manag.

[28] Greenberg D, Siebzehner MI, Pliskin JS (2009). The process of updating the National List of health Services in Israel: is it legitimate? Is it fair? International journal of technology assessment in health care. Cambridge..

[29] Ahn J, Kim G (2012). Hae sun Suh, sang moo Lee. Social values and healthcare priority setting in Korea. J of Health Org and Mgt.

[30] Choe YJ, Han OP, Cho H, Bae G-R, Chun B-C, Kim J-H (2014). Prioritization of the introduction of new vaccines to the national immunization program in the Republic of Korea. Vaccine..

[31] Ashton T, Tenbensel T, Cumming J, Barnett P (2008). Decentralizing resource allocation: early experiences with district health boards in New Zealand. J Health Serv Res Policy.

[32] Defechereux T, Paolucci F, Mirelman A, Youngkong S, Botten G, Hagen TP (2012). Health care priority setting in Norway a multicriteria decision analysis. BMC Health Serv Res.

[33] Waldau S, Lindholm L, Wiechel AH (2010). Priority setting in practice: participants opinions on vertical and horizontal priority setting for reallocation. Health Policy.

[34] Waldau S (2015). Bottom-up priority setting revised. A second evaluation of an institutional intervention in a Swedish health care organisation. Health Policy.

[35] Bate A, Donaldson C, Ray H (2007). Resource allocation in orthopaedics: economic evaluation to priority setting. Clin Orthop Relat Res.

[36] Wilson E, Sussex J, Macleod C, Fordham R (2007). Prioritizing health technologies in a primary care trust. J Health Serv Res Policy.

[37] Airoldi M, Bevan G, Morton A, Oliveira M, Smith J (2008). Requisite models for strategic commissioning: the example of type 1 diabetes. Health Care Manage Scie.

[38] Marsh K, Dolan P, Kempster J, Lugon M (2013). Prioritizing investments in public health: a multi-criteria decision analysis. J Public Health (Oxf).

[39] Goodwin E, Frew EJ (2013). Using programme budgeting and marginal analysis (PBMA) to set priorities: reflections from a qualitative assessment in an English primary care trust. Soc Sci Med.

[40] Airoldi M (2013). Disinvestments in practice: overcoming resistance to change through a sociotechnical approach with local stakeholders. J Health Polit Policy Law.

[41] Holmes RD, Steele JG, Exley C, Vernazza CR, Donaldson C. Use of programme budgeting and marginal analysis to set priorities for local NHS dental services: learning from the north east of England. J Public Health (Oxf). 2018;40(4):e578-e585. 10.1093/pubmed/fdy075. 10.1093/pubmed/fdy075 29726998

[42] Vernazza CR, Taylor G, Donaldson C, Gray J, Holmes R, Carr K (2019). How does priority setting for resource allocation happen in commissioning dental services in a nationally led, regionally delivered system: a qualitative study using semistructured interviews with NHS England dental commissioners. BMJ Open.

[43] Canham-Chervak M, Hooper TI, Brennan FH, Craig SC, Girasek DC, Schaefer RA (2010). A systematic process to prioritize prevention activities: sustaining Progress toward the reduction of military injuries. Am J Prev Med.

[44] Peacock S, Ruta D, Mitton C, Donaldson C, Bate A, Murtagh M (2006). Using economics to set pragmatic and ethical priorities. BMJ..

[45] INAHTA [Internet]. [cited 2018 Jul 12]. Available from: www.inahta.org.

[46] Submission FAQs for Industry - Pharmaceuticals [Internet]. EUnetHTA. [cited 2019 Jul 19]. Available from: https://www.eunethta.eu/frequently-asked-questions-for-the-pharmaceutical-industry/.

[47] Mooney GH, Russell EM, Weir RD (1986). Choices for health care: a practical introduction to the economics of health provision.

[48] Mitton C, Seixas BV, Peacock S, Burgess M, Bryan S (2019). Health technology assessment as part of a broader process for priority setting and resource allocation. Appl Health Econ Health Policy.

[49] Harris C, Green S, Elshaug AG. Sustainability in Health care by Allocating Resources Effectively (SHARE) 10: operationalising disinvestment in a conceptual framework for resource allocation. BMC Health Serv Res. 2017; [cited 2018 Jan 27];17. Available from: https://www.ncbi.nlm.nih.gov/pmc/articles/PMC5590199/. 10.1186/s12913-017-2506-7 PMC5590199 28886740

[50] Daniels N, Sabin J (2002). Setting limits fairly: can we learn to Share medical resources? 1 edition. Oxford.

[51] Daniels N (2000). Accountability for reasonableness. BMJ.

[52] Gibson JL, Singer DKM, PA. Evidence, Economics and Ethics: Resource Allocation in Health Services Organizations: Healthcare Quarterly; 2005. [cited 2017 May 29]. Available from: http://www.longwoods.com/content/17099. 10.12927/hcq..17099 15828568

[53] Edwards RT, Charles JM, Thomas S, Bishop J, Cohen D, Groves S (2014). A national Programme budgeting and marginal analysis (PBMA) of health improvement spending across Wales: disinvestment and reinvestment across the life course. BMC Public Health.

[54] Mooney G (2002). Priority setting in mental health services. Appl Health Econ Health Policy.

[55] Haas M, Viney R, Kristensen E, Pain C, Foulds K (2001). Using programme budgeting and marginal analysis to assist population based strategic planning for coronary heart disease. Health Policy.

[56] Angelis A, Kanavos P (2017). Multiple criteria decision analysis (MCDA) for evaluating new medicines in health technology assessment and beyond: the advance value framework. Soc Sci Med.

[57] Goetghebeur M, Wagner M, Khoury H, Levitt R, Erickson L, Rindress D (2008). PMC50 evidence ANDVALUE: impact on decision making—the EVIDEM framework and potential applications. Value Health.

[58] Airoldi M, Morton A, Smith JAE, Bevan G (2014). STAR—people-powered prioritization: a 21st-century solution to allocation headaches. Med Decis Mak.

[59] Kapiriri L, Razavi D (2017). How have systematic priority setting approaches influenced policy making? A synthesis of the current literature. Health Policy.

[60] Tsourapas A, Frew E (2011). Evaluating ‘success’ in Programme budgeting and marginal analysis: a literature review. J Health Serv Res Policy.

